# An Effective Label-Free Electrochemical Aptasensor Based on Gold Nanoparticles for Gluten Detection

**DOI:** 10.3390/nano12060987

**Published:** 2022-03-17

**Authors:** Rossella Svigelj, Ivan Zuliani, Cristian Grazioli, Nicolò Dossi, Rosanna Toniolo

**Affiliations:** Department of Agrifood, Environmental and Animal Science, University of Udine, Via Cotonificio 108, 33100 Udine, Italy; zuliani.ivan@spes.uniud.it (I.Z.); cristian.grazioli@uniud.it (C.G.); nicolo.dossi@uniud.it (N.D.)

**Keywords:** aptamers, aptasensor, beer, electrochemical impedance spectroscopy, food allergen, gluten, gold nanoparticles, impedimetric biosensor, soy sauce

## Abstract

Nanomaterials can be used to modify electrodes and improve the conductivity and the performance of electrochemical sensors. Among various nanomaterials, gold-based nanostructures have been used as an anchoring platform for the functionalization of biosensor surfaces. One of the main advantages of using gold for the modification of electrodes is its great affinity for thiol-containing molecules, such as proteins, forming a strong Au-S bond. In this work, we present an impedimetric biosensor based on gold nanoparticles and a truncated aptamer for the quantification of gluten in hydrolyzed matrices such as beer and soy sauce. A good relationship between the R_ct_ values and PWG-Gliadin concentration was found in the range between 0.1–1 mg L^−1^ of gliadin (corresponding to 0.2–2 mg L^−1^ of gluten) with a limit of detection of 0.05 mg L^−1^ of gliadin (corresponding to 0.1 mg L^−1^ of gluten). The label-free assay was also successfully applied for the determination of real food samples.

## 1. Introduction

Nanomaterials have been applied in different fields such as electrochemistry [1,2,3], medicine [4,5], engineering [6], and many others [7,8]. The physical and chemical properties of nanomaterials change depending on their composition, shape, and size, and thanks to their tunable characteristics, they are increasingly used to improve the performance of electrochemical sensors [9,10,11]. In addition, their high surface area allows the easy immobilization of biomolecules such as proteins and aptamers; hence, applications based on nanoparticles in biosensor development are growing [12,13,14,15].

Among various metallic nanomaterials, gold-based nanostructures have been used as an anchoring platform for the functionalization of biosensor surfaces [16]. One of the main advantages in the use of gold is its biocompatibility and the strong bond that can be formed between molecules containing thiols, such as proteins, and the surface of gold nanomaterials [17]. Moreover, it is possible to use gold nanomaterials with different shapes such as nanoparticles, nanorods, nanoshells, and nanostars [18]. Gold nanoparticles (AuNPs) present a high surface-to-volume ratio, high surface energy, and the ability to improve electron transfer between redox species and the electrode surface. Hence thanks to their characteristics, the use of nanomaterials for the construction of electrochemical biosensors enhances the analytical performance when compared to other biosensor designs [19,20].

Highly sensitive nanobiosensors make possible the detection of analytes when they are present in very low concentrations, such as food allergens [21,22]. According to the Food and Drug Administration (FDA), about 90% of food allergy cases are triggered by milk, eggs, fish, shellfish, tree nuts, wheat, peanuts, and soy [23]. So far, a diet without food containing allergenic ingredients is the only strategy that avoids allergies in hypersensitive individuals. Gluten is identified as one of the eight major food allergens. Accordingly, the European Union took specific action to protect the health of consumers suffering from coeliac disease, defining that the term “gluten-free” is allowed in foods with a gluten content of less than 20 mg/kg, and that “very low gluten” refers only to products with a quantity of gluten between 20 mg/kg and 100 mg/kg. In predisposed individuals, the intake of gluten causes coeliac disease, which is a chronic condition that involves the immune system and compromises the intestinal villi [24,25]. After ingestion, gluten is partially broken by gastrointestinal enzymes into peptides [26]. These peptides are rich in proline and glutamine, and this characteristic makes them resistant to proteolysis in the human gut [27]. Some of these peptides can cause an immune-toxic response, which may evolve in local tissue damage [28]. These peptides can be found in some fermented foods, such as beer. From an analytical point of view, these types of real matrices are difficult to analyze because the target is small compared to the non-hydrolyzed protein [29,30]. For this reason, label-free assays are important. Unlike the sandwich assays, in which the protein must be present in an intact form, with a label-free approach, only one recognition element is used. Hence, it is possible to carry out a precise quantification of gluten even in matrices where the protein is in the hydrolyzed form. Moreover, the simplicity and rapidity of the detection make label-free assays of increasing interest.

In recent years, various approaches have been proposed for the quantification of gluten based on aptamers [31,32], among them various sandwich assays [33,34] and some label-free [35,36]. However, the label-free assays proposed so far have not been very effective in the determination of gluten at low concentrations, a very important factor for the population of people sensitive even to very low concentrations of gluten. For this purpose, in this work, we present a rapid platform based on electrochemical impedance spectroscopy (EIS) for the quantification of gluten in hydrolyzed matrices such as beer and soy sauce. The proposed biosensor uses AuNPs as an anchoring platform and a truncated aptamer with a high affinity for the analyte [33]. The ability of gold nanoparticles to provide a stable immobilization of biomolecules maintaining their bioactivity will be exploited. Moreover, the benefits of using a nanostructured gold surface instead of a gold surface are an improved electron transfer property and increased surface area for the subsequent modifications. In the literature, some studies have already reported the successful development of biosensors on screen-printed carbon electrodes modified with AuNPs for the determination of analytes present in low concentrations [37,38].

## 2. Materials and Methods

### 2.1. Chemicals and Reagents

5′-tagged (biotin) truncated aptamer Gli4-T (sequence: CTA CAC ATG TCT GAA TGC C) was obtained HPLC-purified from Sigma Aldrich (Milan, Italy). Bovine serum albumin (BSA), sorbitol, and biotin were purchased from Sigma Aldrich (Milan, Italy). Streptavidin and Horseradish peroxidase (HRP) conjugated with streptavidin were purchased from Merck (Milan, Italy). Tetrachloroauric acid was purchased by J.M. Chemicals (London, UK). PWG-Gliadin was kindly provided by the Prolamine Working Group (Freising, Germany). Ultrapure water (R > 18 MΩ) was obtained by means of an Elga Purelab flex 4 system (Veolia Water Technologies, Milan, Italy) and used for the preparation of buffer solutions.

### 2.2. Apparatus

Screen-printed carbon electrodes were purchased by Dropsens (Metrohm, Milan, Italy). All voltammetric and impedance measurements were performed using an Autolab PGSTAT204 potentiostat (Metrohm, Milan, Italy) managed by Nova software version 3.2 and connected to the SPCE by means of a CAC connector cable from Dropsens (Metrohm, Milan, Italy).

Morphological and structural properties of AuNPs were investigated by Field Emission Scanning Electron Microscopy (FE-SEM) performed by JEOL model JSM-7610FPlus.

### 2.3. Electrochemical Impedance Spectroscopy (EIS) Measurements

The electrochemical impedance spectroscopy measurements were conducted using the redox probe [Fe(CN)_6_]^4−^/[Fe(CN)_6_]^3−^ 2 mM each, KCl 3 mM in PBS. The applied potential was 0.115 V (half-wave potential of the redox pair), while the frequency was varied in the range from 10,000 Hz to 0.01 Hz, with an amplitude of 0.005 V. The resistance to charge transfer (R_ct_) was calculated using NOVA software.

All the measurements were carried out at room temperature on an SPCE. The same measurement procedure was also used in the analysis of real samples.

### 2.4. Modification Procedure of SPCE with AuNPs

In order to increase the sensitivity of the impedimetric biosensor, two strategies exploiting gold particles were evaluated. The first strategy involved the modification of the surface by drop-casting. For this purpose, 20 µL of a 5 mM AuNPs aqueous solution, previously synthesized in our laboratory as reported in the literature [39], were drop-casted on the electrode surface and allowed to evaporate. Briefly, the AuNPs were synthesized as follows: 1 mM HAuCl_4_ solution was put to reflux under stirring, then a 38 mM sodium citrate solution was quickly added, the color of the solution changed from yellow to deep red. The system was let on reflux for 20 min, and after that time, the solution was cooled down to room temperature under stirring.

**Figure 1:**
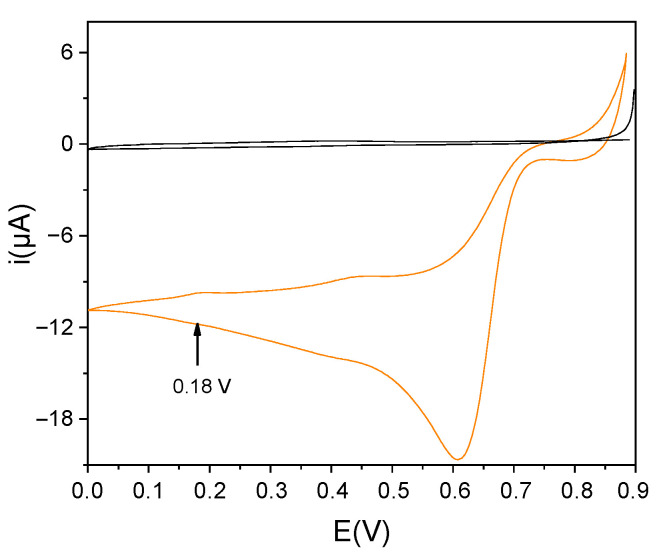
Cyclic voltammetry recorded on SPCE for a 1 mM solution of HAuCl_4_ in 0.5 M H_2_SO_4_ (orange line). Background cyclic voltammetry recorded in 0.5 M H_2_SO_4_ (black line). The arrow indicates the potential chosen for the electrochemical generation of AuNPs.

The second strategy involved the electrochemical deposition of AuNPs. In this approach, two parameters are crucial to controlling the size and shape of NPs: the potential applied and the time of deposition. First, we conducted a voltammetric investigation, as reported in Figure 1. Then, to identify the best conditions, we performed the deposition of AuNPs at different potentials of 0.6 V, 0.4 V, and finally, 0.18 V. As can be seen from Figure S1, the measurements of impedance conducted on the modified SPCE indicate that the surface with the least resistance at the charge transfer is that modified at 0.18 V, in agreement with the literature [10,39].

Secondly, we studied the deposition time, ensuring the best performance. For this purpose, chronoamperometry measurements were carried out at different times (10, 20, 30, 40, 50, 60 s), and as can be seen from Figure S2, the amount of charge expended during the deposition increases with time. Then, EIS measurements for each modification at different times were performed. Figure S3 shows that after 50 s, there is no improvement from the point of view of charge transfer. In the light of these results, all the subsequent experimentation was conducted by modifying the electrodes as follows: 40 µL of a 1 mM solution of HAuCl_4_ in 0.5 M H_2_SO_4_ was placed on the electrode surface, and 0.18 V was applied for 50 s.

### 2.5. Biosensor Assembly

**Figure 2:**
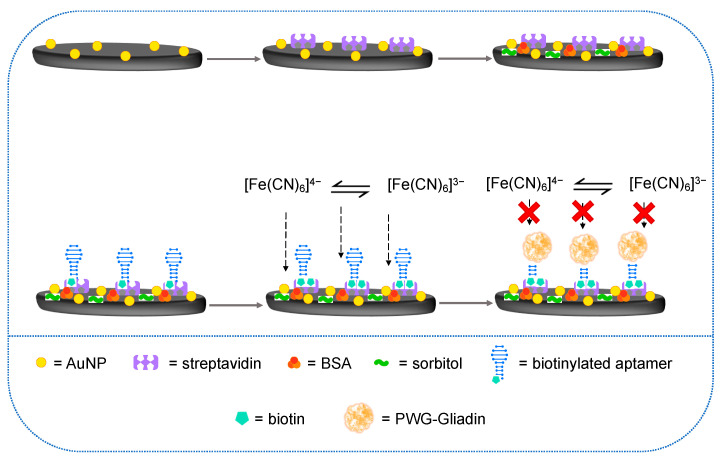
Schematic representation of the aptasensor design and its working principle: after the electrochemical deposition of AuNPs streptavidin was immobilized on the working electrode, then a solution of BSA and sorbitol was used to block the surface; subsequently, the biotinylated aptamer was immobilized, and after a final step of blocking with biotin, the sensor was ready to use. The redox probe [Fe (CN) 6]^3^^−^/[Fe (CN) 6]^4^^−^ was employed to evaluate the presence or absence of PWG-Gliadin.

First, the surface of the working electrode of the SPCE was washed with 500 µL of ethanol and dried with air. Subsequently, the electrochemical deposition of gold nanoparticles (AuNPs) was carried out by drop-casting 40 µL of a solution of tetrachloroauric acid (HAuCl_4_) 1 mM in 0.5 M H_2_SO_4_ and applying 0.18 V for 50 s. Next, the surface of the SPCE was washed three times with phosphate buffer solution (PBS) pH 7.4 and dried with air. A total of 10 µL of streptavidin 1 mg mL^−1^ were immobilized on the bare carbon working electrode by adsorption at 4 °C overnight; then, the surface was blocked with 20 µL of BSA 1% and sorbitol 6% in PBS for 30 min. Then, the SPCE was again washed and covered with 20 µL 1 µM solution of Gli4T-biotin in PBS for 30 min. Finally, the surface was covered with 20 µL of 0.5 µM biotin in PBS for 10 min. Figure 2 shows the functionalization protocol of the modified SPCE with electrogenerated AuNPs, in which, in the end, the presence or absence of PWG-Gliadin was detected using the redox probe [Fe (CN) 6]^3−^/[Fe (CN) 6]^4−^.

### 2.6. Gliadin Detection

20 µL of the solutions at different concentrations of PWG-Gliadin in buffer solution (20 mM Tris-HCl, 100 mM NaCl and 2 mM MgCl_2_) were deposited on the working electrodes of the previously modified SPCEs.

After half an hour of incubation, the surface of the WE of the SPCE was rinsed three times with 250 µL aliquots of buffer and dried with air. Subsequently, 40 µL of a PBS solution containing 3 mM KCl, 2 mM [Fe (CN) 6]^3−^ and 2 mM [Fe (CN) 6]^4−^ were added on the SPCE, and immediately the impedance measurement was performed. The same procedure was also used in the analysis of real samples after dilution and enrichment with known quantities of PWG-Gliadin.

## 3. Results and Discussion

### 3.1. Electrochemical Characterization of SPCE Modified with AuNPs

**Figure 3:**
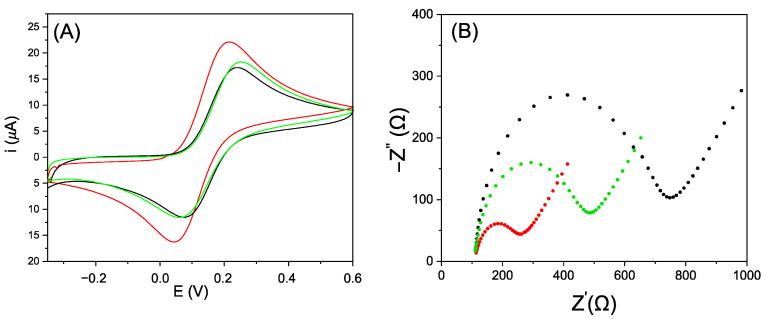
(**A**) Cyclic voltammograms of ferrocyanide 1 mM in KCl 100 mM on the bare screen-printed carbon electrode (SPCE) in black, on the SPCE modified with AuNPs by drop-casting in green and on the SPCE modified with electrogenerated AuNPs in red. (**B**) Nyquist plots recorded using the redox probe [Fe(CN)_6_]^4^^−^/[Fe(CN)_6_]^3^^−^ 2 mM each, KCl 3 mM in PBS at unmodified SPCE (black, R_ct_ = 614.44 Ω), modified with AuNPs deposited by drop-casting (green, R_ct_ = 372.85 Ω) and modified with electrogenerated AuNPs (red, R_ct_ = 172.95 Ω).

In order to evaluate the efficiency of the two modification strategies, the SPCEs were analyzed by cyclic voltammetry and EIS. The results are shown in Figure 3. The voltammetric behavior of the SPCE modified with electrogenerated AuNPs confirms the ability of gold nanoparticles to enhance conductivity. In fact, Figure 3B shows that in all spectra, a semicircular portion is predominant and how both modification strategies lead to a reduction of the related radius. However, by comparing the Nyquist plots obtained on the gold-modified SPCEs, it is possible to see how the electrochemical deposition of AuNPs leads to a lower charge transfer resistance, confirming that this approach is more effective than the modification carried out by drop-casting. To attain more insight concerning these behaviors, a SEM characterization of both modified screen-printed electrodes was performed.

### 3.2. SEM Characterization of Modified SPCE

**Figure 4:**
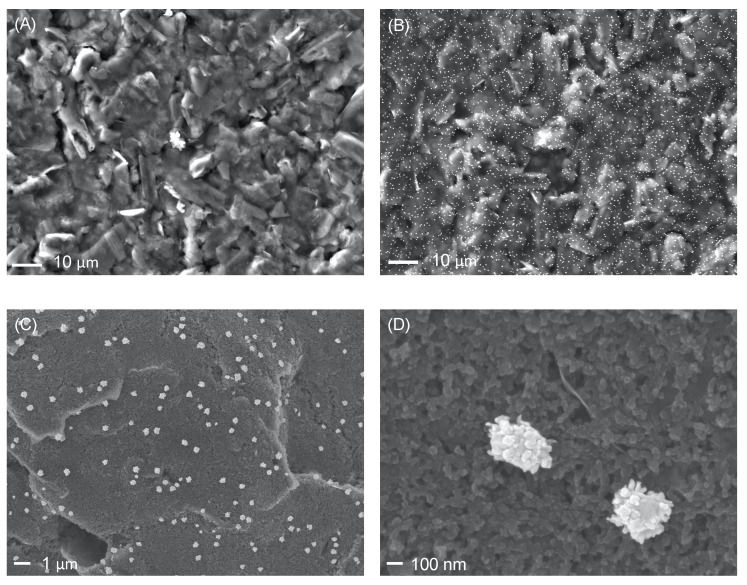
FE-SEM images of unmodified SPCE (**A**), AuNPs-modified SPCE (**B**), AuNPs-modified SPCE at different magnifications (**C**,**D**).

The morphology of SPCEs modified with AuNPs was investigated by a Field Emission Scanning Electron Microscopy. Figure 4A,B shows the SEM images of an SPCE before and after the modification with electrogenerated AuNPs. Instead, in Figure 4C,D, the morphology and size of the AuNPs can be seen in more detail. It is possible to observe that the carbon electrode surface is evenly covered by spherical AuNPs. Conversely, the same investigation performed on SPCE modified by drop-casting led to the formation of AuNPs aggregates mainly located at the outer edges of the carbon electrode and a scarce presence of nanoparticles on the electrode surface, see Figure S4. The images were obtained with an electron beam generated using two different electron acceleration voltages (15 and 5 kV), and the acquisition was carried out using a detector for secondary electrons in the two modes LEI (lower secondary electron detector) and SEI (secondary electron detector). As can be noticed, the electrochemical deposition of AuNPs is a very effective approach leading to a homogeneous and uniform distribution of AuNPs with dimensions between 200 and 400 nm over the entire surface of the SPCE.

### 3.3. Electrochemical Characterization of the Biosensor

**Figure 5:**
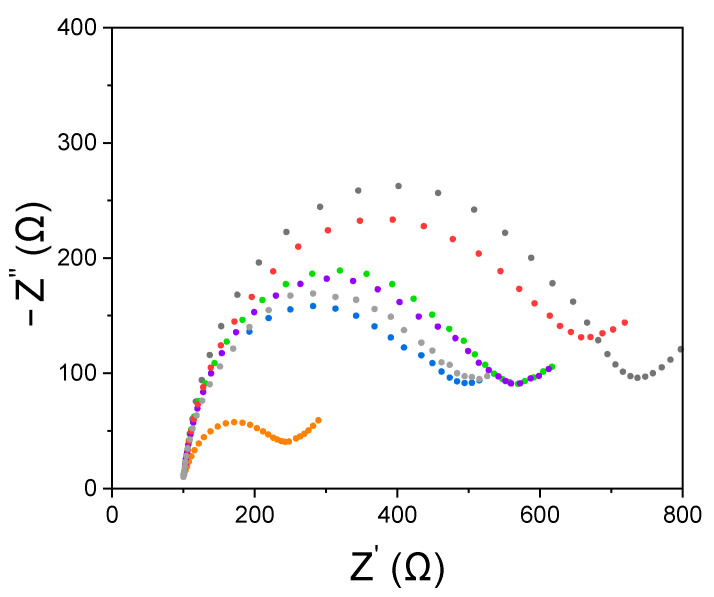
Nyquist plots recorded on SPCE using the redox probe [Fe(CN)_6_]^4^^−^/[Fe(CN)_6_]^3^^−^2 mM each, KCl 3 mM in PBS: unmodified (black, R_ct_ = 614.44 Ω), modified with electrogenerated AuNPs (orange, R_ct_ = 172.95 Ω), modified with streptavidin (blue, R_ct_ = 384.82 Ω), after 30 min of BSA-sorbitol (grey, R_ct_ = 403.68 Ω) after 30 min of Gli4-T (purple, R_ct_ = 456.59 Ω), after 10 min of biotin (green, R_ct_ = 469.33 Ω) and after 30 min of PWG-Gliadin 0.5 mg L^−1^ (red, R_ct_ = 586.61 Ω).

Based on the results reported in Section 3.1 and Section 3.2, the subsequent experimental work has been performed on the SPCE modified with the electrogenerated AuNPs. In order to verify the effectiveness of the SPCE modification procedure, a surface characterization was performed at the end of each modification step using EIS measurements. Figure 5 shows the relevant Nyquist plots confirming the correct surface modifications. In fact, the resistance to charge transfer increase progressively from the deposition of streptavidin (R_ct_ = 384.82 Ω) to BSA-sorbitol (R_ct_ = 403.68 Ω) and Gli4-T (R_ct_ = 456.59 Ω). In addition, after biotin immobilization (R_ct_ = 469.33 Ω), there were no substantial changes indicating that the surface was already properly blocked and ready for subsequent measurements. Impedance behavior recorded at SPCE is due to an increasing difficulty for the electroactive pair ferrocyanide/ferricyanide to reach the electrode surface and to an increasingly resistive electrode surface. As expected, this trend can be correlated to the correct adsorption of biomolecules during the modification steps. Finally, after the incubation with PWG-Gliadin 0.5 mg L^−1^, thanks to steric hindrance, the value of R_ct_ increased substantially (R_ct_ = 586.61 Ω).

### 3.4. Gliadin Quantification by Label-Free Biosensor

**Figure 6:**
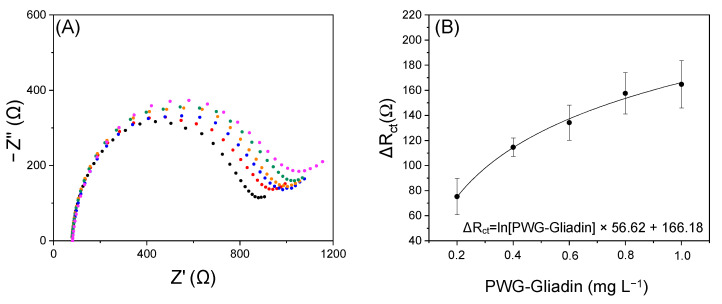
(**A**) Nyquist plots recorded for increasing concentrations of PWG-Gliadin (0, 0.2, 0.4, 0.6, 0.8, 1 mg L^−1^), (**B**) Calibration curve used for the determination of the gliadin content in real samples. The error bars correspond to the standard deviation evaluated on a minimum of three measurements at each point.

The biosensor was calibrated by analyzing solutions with a known concentration of PWG-Gliadin, which is a fraction of gluten (approximately 50%) commonly used as an analytical target. The signals obtained for each concentration of PWG-Gliadin have been subtracted from the blank to compare the data obtained on different days. Figure 6 shows the Nyquist plots recorded for increasing concentrations of PWG-Gliadin (0, 0.2, 0.4, 0.6, 0.8, 1 mg L^−1^) and the relative calibration curve obtained from three different repetitions.

The calibration fitted the Levenberg–Marquardt function with a correlation of 0.994. The limit of detection (LOD), calculated as three times the standard deviation of the blank signal divided by the slope calculated for the linear dynamic range, and the limit of quantification (LOQ), estimated by multiplying the LOD by 3.3, were found to be 0.05 and 0.16 mg L^−1^ of gliadin (corresponding to 0.1 and 0.32 mg L^−1^ of gluten respectively). Stability tests show that the sensor response does not change significantly during 5 days, see Figure S5. Finally, as can be seen from Table 1, the proposed sensor successfully competes with those reported in the literature and present on the market so far.

### 3.5. Real Samples Analysis

The gluten content of two gluten-free beers and one gluten-free soy sauce were tested. In these samples, gluten is present in the hydrolyzed form and, therefore, cannot be determined with the classic sandwich approaches. The samples were also analyzed after their enrichment with a known quantity of PWG-Gliadin. All samples were diluted 1/20 and analyzed following the procedure previously described for PWG-Gliadin standards. Table 2 reports gluten contents determined in real samples, in enriched samples, and the related recovery (%). Some representative raw Nyquist plots of real samples and spiked samples are reported in Figure S6.

As can be seen from Table 2, in all three analyzed samples, the gluten content is below the limit of 20 mg L^−1^. Furthermore, the recoveries calculated for the spiked samples are close to 100%, confirming the aptasensor’s ability to provide accurate and reproducible data in these matrices.

## 4. Conclusions

In this work, we have proposed a simple label-free and easy-to-use biosensor. The impedimetric aptasensor developed was built on disposable graphite screen-printed electrodes modified with gold nanoparticles and the truncated aptamer Gli4-T. The modification of the biosensor surface was carried out by the electrogeneration of AuNPs using tetrachloroauric acid. A good relationship between the R_ct_ values and PWG-Gliadin concentration was found in the range between 0.1–1 mg L^−1^ of gliadin (corresponding to 0.2–2 mg L^−1^ of gluten) with a limit of detection of 0.05 mg L^−1^ of gliadin (corresponding to 0.1 mg L^−1^ of gluten). The label-free assay was also applied for the determination of spiked samples of food bought in local supermarkets. These results can be very important for the improvement of gluten quantification, especially in those matrices where gluten is present in the hydrolyzed form. Finally, our study demonstrates that the use of gold particles is a valid strategy to increase the sensitivity of label-free assays.

## Figures and Tables

**Table 1:** Comparison of the analytical performance of different label-free approaches for gluten detection.

Method	Recognition Element	LOD/mg L^−1^ (Gluten)	Linear Range/mg L^−1^ (Gluten)	Ref.
Label-free (impedance)	aptamer	5	-	[35]
Label-free (impedance)	antibody	5	5–20	[36]
Label-free (impedance)	antibody	14	0–20	[40]
Label-free (impedance)	aptamer	0.1	0.4–2	This work

**Table 2:** Gluten quantification in different food matrices with the label-free aptasensor.

Sample	Expected Gluten Concentration (mg L^−1^) *	Gluten Concentration (mg L^−1^) **	Recovery (%) ***
Beer 1	<20	12.96 ± 1.68	\
Beer 1 (spiked with 5 mg L^−1^)	16.66	16.80 ± 2.21	101
Beer 2	<20	18.38 ± 1.04	\
Beer 2 (spiked with 10 mg L^−1^)	26.54	27.81 ± 3.49	105
Soy sauce	<20	8.72 ± 0.27	\
Soy sauce (spiked with 10 mg L^−1^)	17.85	16.59 ± 2.02	93

* The expected concentration in the added samples was calculated considering the initial concentration, the addition of PWG-Gliadin multiplied by two and the dilution attributable to the addition itself. ** Average of three repetitions. *** Obtained as the ratio between the gluten concentration determined and that expected in the spiked samples.

## Data Availability

The data presented in this study are available on request from the corresponding author.

## Supplementary Materials

The following supporting information can be downloaded at: https://www.mdpi.com/article/10.3390/nano12060987/s1, Figure S1: Characterization of the electrode surface with EIS after the electrodeposition of AuNPs at different potentials (0.18 V, 0.4 V and 0.6 V); Figure S2: Trend of the charge (C) spent during the electrogeneration of AuNPs at 0.18 V at different times (10, 20, 30, 40, 50 and 60 s); Figure S3: Characterization of the electrode surface with EIS after the electrodeposition of AuNPs at different times (10, 20, 30, 40, 50 and 60 s); Figure S4: (A) Enlargement of the surface of the SPCE where most of the drop-casted nanoparticles form aggregates. (B) FE-SEM images of the carbon surface obtained by drop-casting of AuNPs; Figure S5: Biosensor stability experiment: ∆R_ct_ (Ω) values recorded in different days for a solution containing 0.6 mg L^−1^ of PWG-Gliadin; Figure S6: Nyquist plots recorded for real samples and their corresponding spiked sample (A) Beer 1, (B) Beer 2 and (C) Soy sauce.
