# Supplementary material for: An Effective Label-Free Electrochemical Aptasensor Based on Gold Nanoparticles for Gluten Detection
Source: Nanomaterials (Basel). 2022 Mar 17;12(6):987. doi: 10.3390/nano12060987 (PMC8953296; doi:10.3390/nano12060987)
Supplement: Supplementary file 1 [file nanomaterials-12-00987-s001.zip › nanomaterials-1614647-supplementary.pdf]

nanomaterials

An effective label-free electrochemical aptasensor based on gold nanoparticles for gluten detection

Rossella Svigelj[1,*], Ivan Zuliani[1], Cristian Grazioli[1], Nicolò Dossi[1], Rosanna Toniolo[1,*]

[1] Department of Agrifood, Environmental and Animal Science, University of Udine, via Cotonificio 108, 33100, Udine, Italy;

* Correspondence: R.S. rossella.svigelj@uniud.it; R.T. rosanna.toniolo@uniud.it

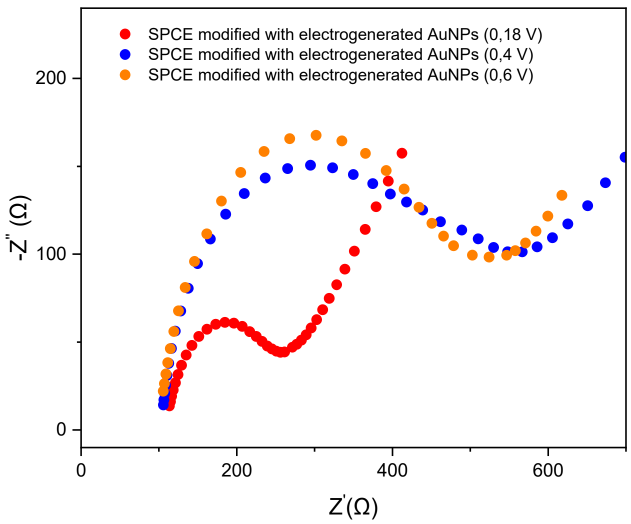

Figure S1 Characterization of the electrode surface with EIS after the electrodeposition of AuNPs at different potentials (0,18 V, 0,4 V and 0,6 V).

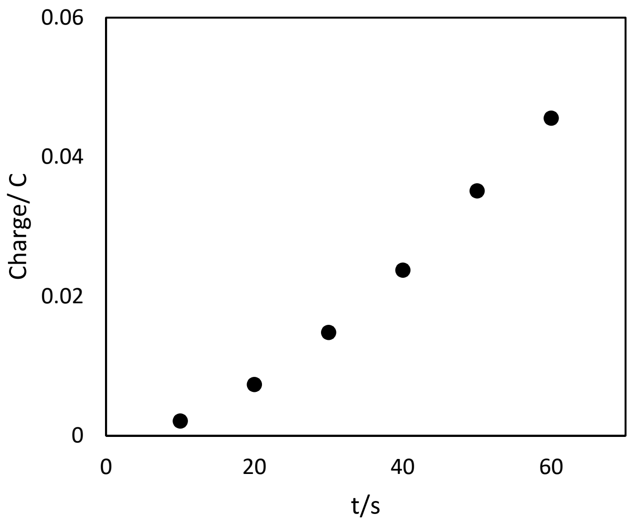

Figure S2 Trend of the charge (C) spent during the electrogeneration of AuNPs at 0,18 V at different times (10, 20, 30,40, 50 and 60 s).

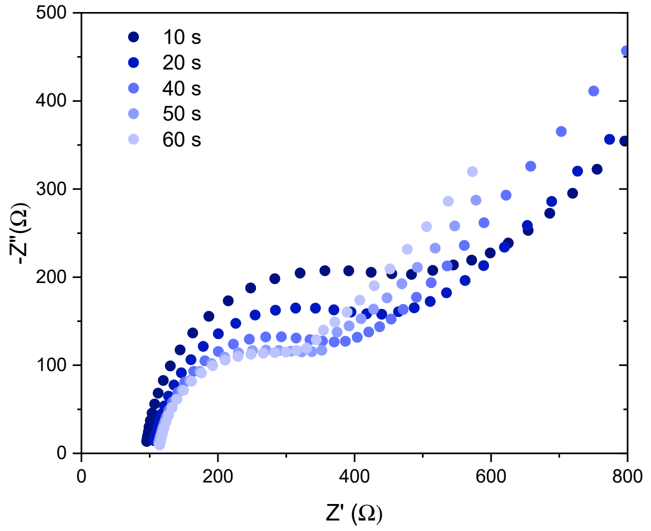

Figure S3 Characterization of the electrode surface with EIS after the electrodeposition of AuNPs at different times (10, 20, 30, 40, 50 and 60 s).

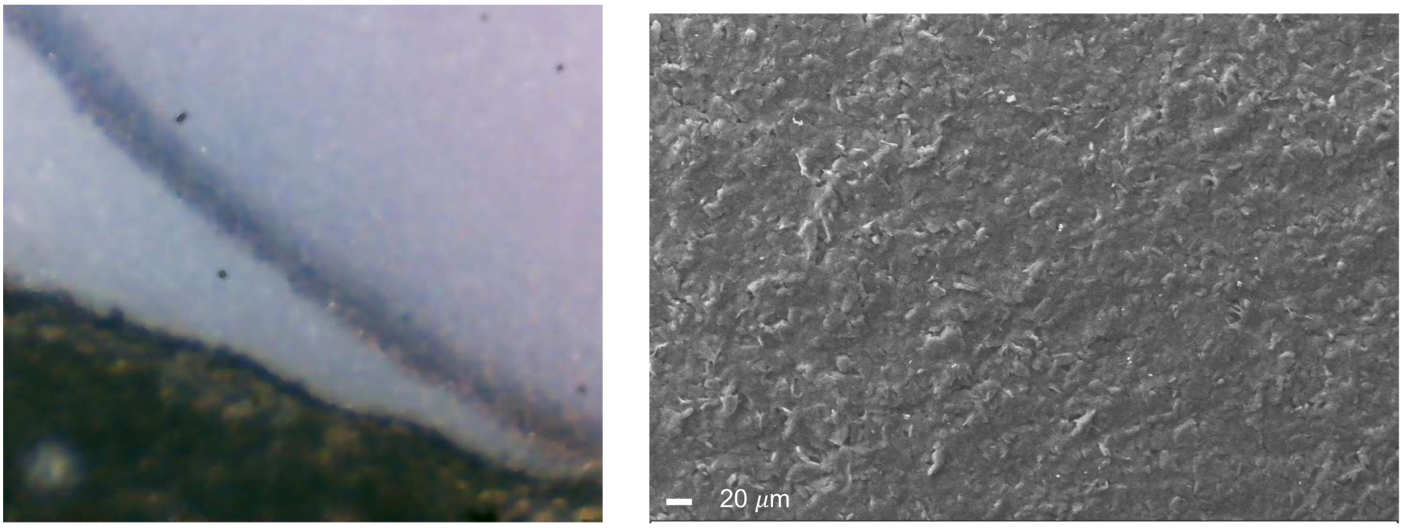

Figure S4 (A) Enlargement of the surface of the SPCE where most of the drop-casted nanoparticles form aggregates. (B) FE-SEM images of the carbon surface obtained by drop-casting of AuNPs.

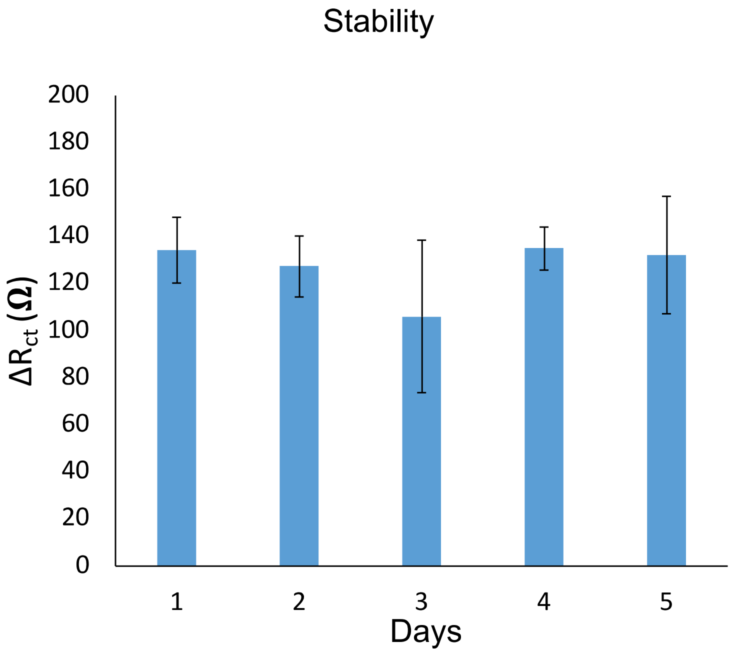

Figure S5 Biosensor stability experiment: ΔR_{ct} (**Ω**) values recorded in different days for a solution containing 0.6 mg L^{-1} of PWG-Gliadin.

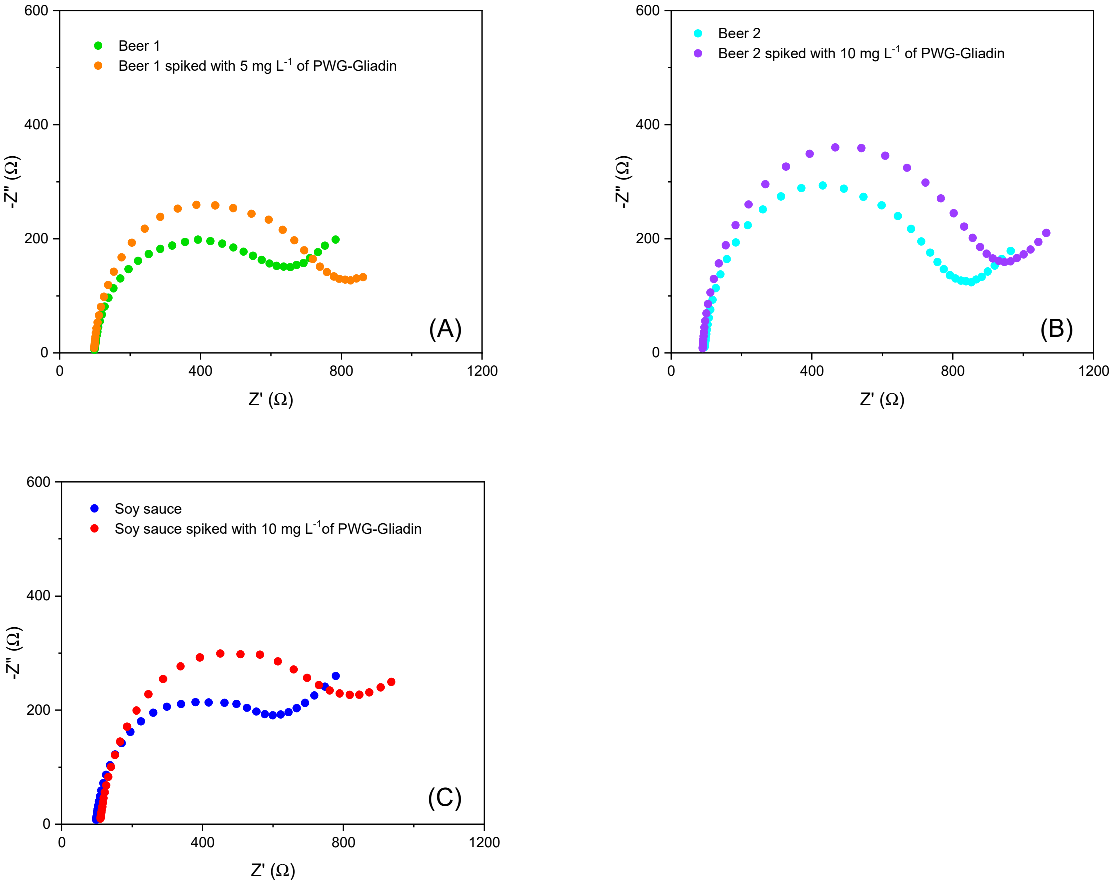

Figure S6 Nyquist plots recorded for real samples and their corresponding spiked sample (A) Beer 1, (B) Beer 2 and (C) Soy sauce.
